# Actinium-225/Bismuth-213 as Potential Leaders for Targeted Alpha Therapy: Current Supply, Application Barriers, and Future Prospects

**DOI:** 10.3390/cancers17183055

**Published:** 2025-09-18

**Authors:** Mohamed F. Nawar, Adli A. Selim, Basma M. Essa, Alaa F. El-Daoushy, Mohamed M. Swidan, Claudia G. Chambers, Mohammed H. Al Qahtani, Charles J. Smith, Tamer M. Sakr

**Affiliations:** 1 Department of Chemistry, Biochemistry and Pharmaceutical Sciences (DCBP), Faculty of Science, University of Bern, CH-3012 Bern, Switzerland; mohamed.nawar@unibe.ch (M.F.N.); alaa.eldaoushy@unibe.ch (A.F.E.-D.); 2 Radioactive Isotopes and Generator Department, Hot Labs Center, Egyptian Atomic Energy Authority, Cairo P.O. Box 13759, Egypt; basmamohamed24@yahoo.com; 3 Labeled Compounds Department, Hot Labs Center, Egyptian Atomic Energy Authority, Cairo P.O. Box 13759, Egypt; adli_a_selim@yahoo.com (A.A.S.); dr_swidan@yahoo.com (M.M.S.); 4 Department of Chemistry, University of Missouri, Columbia, MO 65201, USA; cgcgh4@umsystem.edu; 5 Molecular Imaging and Theranostics Center, University of Missouri, Columbia, MO 65201, USA; 6 Research Division, Harry S. Truman Memorial Veterans’ Hospital, Columbia, MO 65201, USA; 7 Cyclotron and Radiopharmaceuticals Department, King Faisal Specialist Hospital and Research Center, Riyadh 11211, Saudi Arabia; mo1500@hotmail.com; 8 University of Missouri Research Reactor Center, University of Missouri, Columbia, MO 65201, USA; 9 Department of Radiology, School of Medicine, University of Missouri, Columbia, MO 65201, USA

**Keywords:** Actinium-225, Bismuth-213, targeted alpha therapy, radionuclide production, radionuclide generator, radiolabeling techniques, cancer theranostics

## Abstract

Alpha therapy (TAT) relies on combining alpha-emitting radionuclides with specific cell-targeting vectors to deliver a high payload of cytotoxic radiation capable of destroying tumor tissues. TAT efficacy comes from the tissue selectivity of the targeting vector, the high linear energy transfer (LET) of the radionuclide, and the short range of alpha particles in tissues. Recent research studies have been directed to evaluate TAT on a preclinical and clinical scale, including evaluating damage to tumor tissues with minimal toxic radiation effects on surrounding healthy tissues. This review highlights the use of Actinium-225/Bismuth-213 radionuclides as promising candidates for TAT. Herein, we begin with a discussion on the production and supply of [^225^Ac]Ac/[^213^Bi]Bi followed by the formulation of [^225^Ac]Ac/[^213^Bi]Bi-radiopharmaceuticals using different radiolabeling techniques. Finally, we have summarized the preclinical and clinical evaluation of these potential radiotheranostic agents.

## Simple Summary

This review assesses the potential of Actinium-225 and Bismuth-213 as leading radionuclides for targeted alpha therapy, highlighting their ability to deliver potent cytotoxic radiation with high tumor specificity. It addresses current challenges related to their production and radiolabeling, and provides a comprehensive overview of recent preclinical and clinical evaluations demonstrating their promise as effective radiotheranostic agents.

## 1. Introduction

Targeted Alpha Therapy (TAT) offers an innovative approach to cancer treatment. The novel technique of pairing alpha-emitting radionuclides with specific targeting agents, including antibodies or peptides, offers an effective means of treating metastatic tumors. This unique strategy provides a direct cytotoxic radiation dose to targeted cancer cells [1,2,3,4,5,6,7,8].

The use of alpha-emitting radionuclides for cancer treatment has been explored over different conventional methods. This may be attributed to two specific reasons. First, the short penetration length of alpha particles inside the human body (˂0.1 mm) dramatically helps to affect only the targeted cancer cells, protecting the neighboring healthy tissues. Secondly, the high linear energy transfer (LET) of high-energy alpha radiation results in highly effective cell destruction [9,10]. Consequently, alpha-emitters can be more effective in killing metastatic cells that do not respond to other treatment strategies, such as beta- and gamma-emitting irradiations and/or chemotherapeutic medications. As a result, TAT creates a better therapeutic opportunity for cases resistant to conventional therapies [11,12,13,14,15,16]. In this regard, the development of radiopharmaceuticals incorporating alpha-emitting radionuclides for TAT has become a competitive interest for both academic research and commercial industries [17,18,19,20].

Recently, two alpha emitters have been introduced as promising radionuclides for cancer treatment. These radionuclides are [^225^Ac]Ac and its daughter [^213^Bi]Bi, which are a result of the ^233^U decay chain. Despite their potential, several challenges face their optimum usage on a large scale. For example, the worldwide supply of [^225^Ac]Ac is limited to approximately 63 GBq (1.7 Ci) annually. This limited amount of radionuclide can only sustain the treatment of 100–200 patients per year on the global stage. Moreover, tedious post-irradiation separation and purification steps are required to isolate [^225^Ac]Ac from any undesired co-produced isotopes during the production process. Furthermore, the notable lack of a clear understanding of the chemistry of [^225^Ac]Ac has dramatically decreased the chances of producing highly stable, radiolabeled cell targeting agents in an acceptable yield with sufficient pharmacodynamic properties. The daughter of [^225^Ac]Ac, [^213^Bi]Bi, can be produced on-site from the [^225^Ac]Ac/[^213^Bi]Bi generator. However, this daughter radionuclide shares some of the same production challenges as its parent [21].

The primary goal of this article is to provide an in-depth assessment of the production techniques of [^225^Ac]Ac and [^213^Bi]Bi that have been made commercially available for TAT. In addition, we discuss new supply routes that could have the potential to elevate the roles of [^225^Ac]Ac and [^213^Bi]Bi in various medical applications. Moreover, this work highlights the chemistry, the current radiolabeling strategies, and recent biological studies of [^225^Ac]Ac and [^213^Bi]Bi radiopharmaceuticals.

## 2. Chemistry and Radiochemistry of [^225^Ac]Ac and [^213^Bi]Bi

Actinium is the first element in the actinide series. Its electron configuration is [Rn] 6d^1^ 7s^2^, and it usually exists as the Ac^3+^ cation with the configuration of [Rn] 6d^0^ 7s^0^. There are no electrons in the outer shell, making actinium invisible to most forms of spectroscopy, consequently making its characterization difficult [22]. Actinium exists in an aqueous solution in the most stable state (+3), and is hydrolyzed at pH ranges from 8.6 to 10.4 [23]. Ac^3+^ polarizes the coordinated water molecule, affecting the proton release to form [Ac(OH)_3-x_]^x+^. Hence, in radiolabeling investigations, higher radiochemical yields (RCY) are expected when using alkaline buffers due to a decrease in [Ac(OH)_3-x_]^x+^ formation [24]. The chemical behavior of Ac^3+^ is very similar to lanthanum (La^3+^), and La^3+^ is often used as a suitable inactive surrogate for Ac^3+^. Because of a large ionic radius of 1.12 Å and high coordination number, [^225^Ac]Ac is often complexed with large polydentate chelating ligands. However, because of this larger radius, Ac^3+^ forms a weaker electrostatic bond with the donor atoms of the complexing ligand, which may result in instability [25]

**Figure 1:**
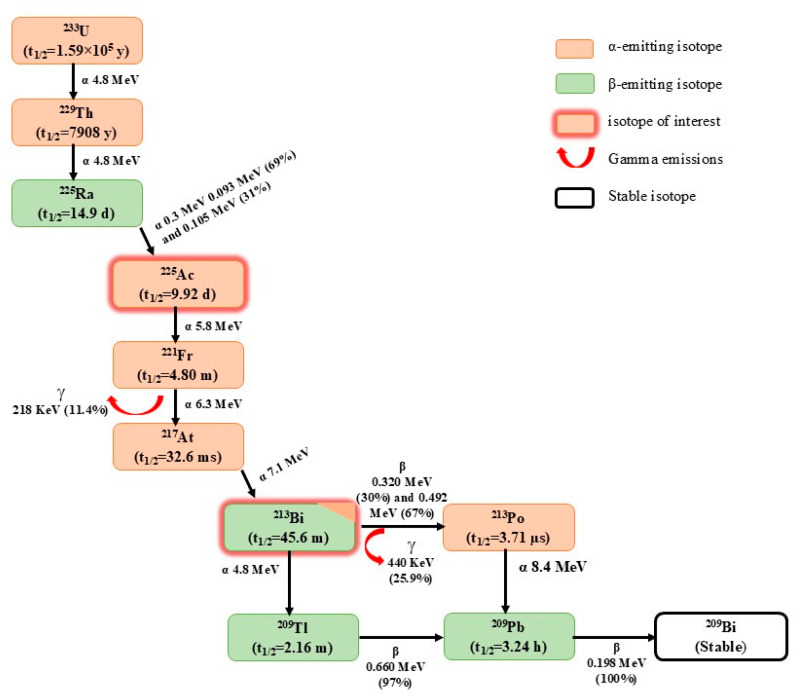
^233^U decay chain.

The impetus driving [^225^Ac]Ac to be used as a radiotherapeutic isotope results from the inherent physical characteristics that it possesses. [^225^Ac]Ac is an alpha-emitting radionuclide known for its relatively long half-life (t_1/2_ = 9.92d) [26]. It decays via the emission of six subsequent daughter radionuclides to reach the most stable long-lived [^209^Bi]Bi (t_1/2_ = 2.01 × 10^19^ y). These daughters result from four alpha (α) decays with energies ranging between 5.8 MeV and 8.4 MeV and two beta (β^−^) decays with energies of 0.093 and 0.660 MeV, which present ^225^Ac as a radiotherapeutic radioisotope as shown in Figure 1 [10].

The co-emission of gamma (γ) rays is also generated within the [^225^Ac]Ac decay chain, which is the result of the decay of [^221^Fr]Fr (218 keV, 11.6% emission probability) and [^213^Bi]Bi (440 keV, 26.1% emission probability). These gamma rays are useful for in vivo imaging applications.

The electron configuration of [^213^Bi]Bi is [Xe] 4f^14^ 5d^10^ 6s^2^ 6p^3^. It forms complexes with coordinating ligands via the three electrons in 6p. Hence, the oxidation state of [^213^Bi]Bi is predominantly +3. In some cases, however, the +5 oxidation state may be seen, as in the case of the two electrons of 6s also participating in bonding. The coordination number of [^213^Bi]Bi ranges from three to ten. Bi^+3^ is a hard Lewis acid, so it has a high affinity for nitrogen and oxygen donors. Yet, complexes with halogens and sulfur are also well known.

[^213^Bi]Bi is a short-lived radionuclide (t_1/2_ = 45.6 min) and emits both alpha and beta particles. It decays mainly by the emission of beta particles to produce the ultra-short-lived and pure alpha emitter, [^213^Po]Po (t_1/2_ = 3.71 µs and E_α_ = 8.4 MeV), with a branching ratio of 97.8%. The residual 2.2% of [^213^Bi]Bi disintegrates with the emission of alpha particles to produce [^209^Tl]Tl with E_α_ = 5.549 MeV (0.16%) and E_α_ = 5.869 MeV (2.01%). Both [^213^Po]Po and [^209^Tl]Tl decay to yield [^209^Pb]Pb (t_1/2_ = 3.24 h), which in turn decays by beta particles to produce long-lived [^209^Bi]Bi (t_1/2_ = 1.9 × 10^19^ y). [^213^Po]Po decays by emitting an 8.4 MeV alpha particle with a penetrating power of 85 µm in human tissue. That makes it responsible for greater than 98% of the emitted alpha particle energy by the decay of [^213^Bi]Bi and is believed to be responsible for its cytotoxic effects. Approximately 92.7% of the overall particle energy that is generated from [^213^Bi]Bi decay results from alpha decay. Simultaneously, the remaining 7.3% of the decay energy results from beta decay [10]. [^213^Bi]Bi also decays by emitting gamma rays with a photon energy of 440 keV, which facilitates the detection of [^213^Bi]Bi biodistribution using a SPECT gamma scintigraph and also for performing pharmacokinetic and dosimetric studies [27].

## 3. Production Techniques of [^225^Ac]Ac

### 3.1. Current [^225^Ac]Ac Sources from Thorium Generators

Currently, [^229^Th]Th generators are considered the primary source of [^225^Ac]Ac production, and they can supply pure [^225^Ac]Ac free from any other actinium isotopes. These generators are being milked to produce hundreds of mCi of [^225^Ac]Ac on a monthly basis. The idea is built on the generation of [^229^Th]Th from the decay of ^233^U hoards, followed by the decay of [^229^Th]Th to [^225^Ra]Ra. Finally, [^225^Ra]Ra decays to produce [^225^Ac]Ac. Most [^233^U]U was produced in the period between 1954 and 1970 through the neutron activation of [^232^Th]Th, which was produced to test its utilization in nuclear reactors and the manufacturing of nuclear weapons. Since 1995, [^229^Th]Th has been extracted from [^233^U]U hoards that were stocked at four main places:

1. The US Department of Energy, Oak Ridge National Laboratory (ORNL) in Oak Ridge, TN, USA: 704 mg of [^229^Th]Th with a radioactivity level of 5.55 GBq (150 mCi) [28].
2. The Directorate for Nuclear Safety and Security of the JRC of the European Commission in Karlsruhe, Germany (formerly known as Institute for Trans-Uranium Elements (ITU) in Karlsruhe, Germany: this source consists of 215 mg of [^229^Th]Th and produces 1.7 GBq (46 mCi) [11,29].
3. Lipinski Institute for Physics and Power Engineering (IPPE) in Obninsk, Russia: this site contains 704 mg of [^229^Th]Th, which has a radioactivity level of 5.55 GBq (150 mCi).
4. At the Belgian Nuclear Research Centre (SCK CEN) in Mol, Belgium, very pure sources of [^229^Th]Th were identified, processed, and used for pre-clinical studies [30].

These four locations are considered to be the primary sources of the worldwide [^225^Ac]Ac supply and produce approximately 63 GBq (1.7 Ci) of [^225^Ac]Ac annually [31,32,33]. The market share of [^225^Ac]Ac between ORNL (USA), ITU (Germany), and IPPE (Russia) is 26.6 GBq (720 mCi), 13.1 GBq (350 mCi) [34], and 26.6 GBq (720 mCi), respectively. In addition, IPPE in Russia does not supply [^225^Ac]Ac regularly [35]. Accordingly, this small amount of [^225^Ac]Ac is sufficient for only 100–200 patient treatments per year. Moreover, on one hand, this low [^225^Ac]Ac production does not satisfy the researcher’s needs or those of increasing clinical trials, which are estimated to require approximately 185 GBq (5 Ci) every year [12,20]. On the other hand, for the development of new [^213^Bi]Bi-based radiopharmaceutical applications, the demand for [^225^Ac]Ac will be even greater.

### 3.2. Developed [^225^Ac]Ac Production Methods

Various methods for large-scale production of [^225^Ac]Ac have been explored, including spallation of natural uranium targets with energetic protons, as well as irradiation of [^226^Ra]Ra targets. The most advanced method is the spallation of [^232^Th]Th, successfully demonstrated at the Institute for Nuclear Research in Russia and Los Alamos National Laboratory in the United States. Routine production of [^225^Ac]Ac via this method has been established under the US Department of Energy’s Tri-Lab initiative, which encompasses Oak Ridge National Laboratory, Brookhaven National Laboratory, and the Los Alamos National Laboratory. Irradiations are conducted at Brookhaven (200 MeV at 165 mA) and Los Alamos (100 MeV at 275 mA), with processing and distribution of the final product occurring at ORNL. A key limitation of this process is the coproduction of long-lived [^227^Ac]Ac, with a half-life of 21.8 years, at activity levels of 0.1% to 0.2% at the end of bombardment (EOB) [36,37,38]. Thus, the vast growth of [^225^Ac]Ac utilization has promoted the exploration of new production techniques. These methods were proposed and tested experimentally, including the production of [^225^Ac]Ac either from [^226^Ra]Ra, or from [^232^Th]Th targets in nuclear reactors and electron or proton accelerators, according to but not limited to the following nuclear reactions:

^226^Ra(γ,n)^225^Ra → ^225^Ac (electron accelerator; electrons of ~25–30 MeV) [35,37,38,39].
^226^Ra(p,2n) ^225^Ac (proton accelerator; low-energy protons ~16 MeV) [37,40,41].
^226^Ra(n,2n)^225^Ra → ^225^Ac (nuclear reactor; high-energy neutrons >6.4 MeV) [37,40,41].
^232^Th(p,x)^225^Ac (Thorium spallation; high-energy protons > 70 MeV) [37,42].
^238^U(p,x)^225^Ac (Uranium spallation; high-energy protons >100 MeV) [37,38].

#### 3.2.1. [225Ac]Ac Production from [^226^Ra]Ra Targets

##### [^225^Ac]Ac Production via Electron Accelerators

Generally, a linear electron accelerator (linac) employs incident electron beams on tungsten targets to generate bremsstrahlung x-rays, which can be used for an external radiation beam therapy. Then, [^226^Ra]Ra needles are subjected to the generated x-ray beams to produce [^225^Ra]Ra (t_1/2_ = 14.9 d), which decays to give [^225^Ac]Ac according to the following designed and experimentally tested photonuclear reaction [29,37,43]:

^2^^26^ Ra( γ,n )^225^Ra → ^225^ Ac

Hence, 20 mg of [^226^Ra]Ra is placed at a distance of 12.5 cm from the bremsstrahlung target. It is then irradiated for one hour using 18 MeV bremsstrahlung x-rays and with an average current of 26 µA [44], resulting in the production of 2.44 MBq (66 µCi) of [^225^Ac]Ac. Based on experimental results, [^225^Ac]Ac production can reach 48 GBq (1.3 Ci) per month using 1 g of [^226^Ra]Ra target. In this regard, different electron accelerators—for instance, the Advanced Rare Isotope Laboratory (ARIEL) facility at TRI-University Meson Facility (TRIUMF, Vancouver, BC, Canada) in Canada and Accélérateur Linéaire et Tandem à Orsay (ALTO) in France—can be used for the production of [^225^Ac]Ac using this technique.

ARIEL is used as an Isotope Separation On-Line (ISOL) facility for radioisotope production via a photofission reaction at 35 MeV and an electron beam of 10 mA. It is mainly for research requirements rather than for medical utilization [45]. The experimental measurements show the possibility of producing 74 TBq (2000 Ci) of [^225^Ac]Ac per month with the irradiation of 1 g of [^226^Ra]Ra target at a higher current and with the use of a different irradiation geometry. Nonetheless, the main challenge that faces production via this procedure is the difficulty for the irradiated target to bear a 100 kW beam, as the available ARIEL ISOL targets can only survive up to 50 kW. In ALTO, theoretically, [^225^Ac]Ac can be produced with a radioactivity level of approximately 56 GBq (1.5 Ci) per month with the use of an electron energy of 50 MeV and 10 A as a beam current [45].

The main advantages of this production method are the generation of pure [^225^Ac]Ac radionuclide and the absence of co-production of any other actinium isotopes. [^224^Ra]Ra is a production by-product and will decay to inert [^220^Rn]Rn (t_1/2_ = 55.6 s). Moreover, the production yield can be elevated with better optimization of the irradiation parameters. However, the large-scale production of [^225^Ac]Ac by medical linacs still faces some difficulties, such as high cost and the lack of suitable infrastructure [39].

##### [^225^Ac]Ac Production via Proton Accelerators

Production of [^225^Ac]Ac by low-energy proton accelerators was first proposed in 2005 according to the nuclear reaction ^226^Ra(p,2n)^225^Ac [46]. This reaction is characterized by its high cross-section (710 mb) at 16.8 MeV, which facilitates its application at various low-energy cyclotrons that are already available worldwide and used to produce a large number of medical radioisotopes. Globally, over 550 cyclotrons have energy greater than 16 MeV, and many of them can be operated at a higher beam current up to 500 µA [47]. Based on the fact that the proton bombardment of [^226^Ra]Ra targets possesses a high cross-section, this approach can be used for large-scale production of [^225^Ac]Ac, which can cover the prospective future medical demand of [^225^Ac]Ac with the sole use of one production site. Theoretically, 4 TBq (108 Ci) of [^225^Ac]Ac can be produced monthly by irradiating 1 g of [^226^Ra]Ra target with a single 20 MeV, 500 µA proton beam [46]. This route does not generate [^227^Ac]Ac (t_1/2_ = 21.77 y) as a side-product, which is a highly toxic actinium isotope. Furthermore, while the (p,n) reaction is expected to produce some [^226^Ac]Ac, the co-production measurements of [^226^Ac]Ac have not been reported [46]. A simple experiment using the FLUKA model shows the appearance of [^226^Ac]Ac (t_1/2_ = 29 h) at the end of bombardment (EOB) with an activity level close to 11% of the total [^225^Ac]Ac yield. Nevertheless, this undesired amount of [^226^Ac]Ac is expected to decrease with time due to the differences in their half-lives [48]. Moreover, the co-production of [^225^Ra]Ra through the ^226^Ra(p,pn)^225^Ra reaction is negligible at optimal energies designed for direct [^225^Ac]Ac production.

##### [^225^Ac]Ac Production via Nuclear Reactors

Lately, nuclear reactors have become the primary source for medical radioisotope production [49]. Nonetheless, [^225^Ac]Ac production using nuclear reactors is still limited. They can be employed for the production of ^225^Ra by bombarding [^226^Ra]Ra targets with high-energy neutrons (>6.4 MeV) according to the following nuclear reaction.

^226^Ra(n,2n)^225^Ra

However, these high-energy neutrons are only present at the tail of the neutron energy spectrum in a typical breeder reactor. Calculated estimations indicate the possibility of producing MBq to GBq (mCi to Ci) quantities of [^225^Ac]Ac every month through the irradiation of 1 g of [^226^Ra]Ra target at a single reactor facility [50]. However, it is worth mentioning that the presence of lower-energy neutrons may result in the co-production of [^227^Ra]Ra, which, because of its toxic effects, can limit the practical use of [^225^Ac]Ac as a medical radioisotope. Moreover, the low [^225^Ra]Ra outcome is not satisfactory, especially with the high cost and the difficulties of safe handling of large [^226^Ra]Ra targets. Due to these critical difficulties, this method has not been experimentally implemented as of yet.

##### Challenges Encountered in the Use of Radium Targets

A profitable radioisotope production procedure strongly necessitates a stable, naturally occurring target material, or even a long-lived isotope, to cover the production cost. For this reason, long-lived [^226^Ra]Ra radionuclide (t_1/2_ = 1600 y) was chosen as a target material for [^225^Ac]Ac supply in all previously mentioned production techniques.

Despite the potential impact of using [^226^Ra]Ra targets, their utilization is accompanied by considerable challenges. These limitations are related to its abundance and underlying safety measures that complicate the production, processing, irradiation, and recycling of [^226^Ra]Ra targets. The handling of [^226^Ra]Ra targets is strongly associated with a number of safety hazards, as [^226^Ra]Ra is a highly radiotoxic isotope, very reactive with both air and water, and its decay generates the gaseous [^222^Rn]Rn daughter radionuclide [51]. Moreover, radium targets are most commonly used as sealed radioactive sources (SRS) wrapped in platinum. The [^226^Ra]Ra decay chain involves five subsequent alpha decays that produce helium, which allows gaseous build-up inside the [^226^Ra]Ra sealed source and may result in source rupture. In addition, high radiation exposure doses are estimated at 8.1 mSv/h at 1 m from 37 GBq (1 Ci, 1 g) of [^226^Ra]Ra, which are caused by hard gamma rays that are also emitted. These limitations represent the primary roadblocks towards the regular use of [^226^Ra]Ra targets.

In 1996, the International Atomic Energy Agency (IAEA) set some regulations for the disposal of [^226^Ra]Ra sources in protracted geological repositories [51]. These regulations strongly restricted the attainability of large [^226^Ra]Ra quantities. To cover the increasing [^225^Ac]Ac demand, new [^226^Ra]Ra sources are required, which may be excavated from the waste of uranium quarrying processes. Nearly 257 mg of [^226^Ra]Ra can be extracted from one ton of uranium ore, which could be upgraded to 12.85 kg of [^226^Ra]Ra annually.

The optimum use of [^226^Ra]Ra sources for a large-scale [^225^Ac]Ac production still requires far more infrastructure than that applied for routine medical isotope production. In addition, as previously mentioned, the necessary safety measures for production would require complementary infrastructure during [^226^Ra]Ra irradiation and processing.

#### 3.2.2. [^225^Ac]Ac Production Using High Energy Proton Spallation of Thorium

The production of [^225^Ac]Ac can alternatively be achieved by the irradiation of thorium metal targets with proton energies greater than 70 MeV and, in some cases, >100 MeV [38,52], according to the following nuclear reaction.

^232^ Th(p,x)^225^Ac

This technique has proposed yields of micro- to milli-Curie quantities of [^225^Ac]Ac. These results have been verified by the Institute for Nuclear Research of the Russian Academy of Sciences (INR), American and Russian research groups at Brookhaven National Laboratory (BNL), and the Los Alamos National Laboratory (LANL) [31,32,35,41,42,53,54,55]. Compared to [^226^Ra]Ra targets, thorium is more radiologically safe and more widely available as a target material. Different thorium sources are currently available, as several countries produce tens of kilograms of thorium. Rare earth mining provides hundreds of tons of thorium annually as a by-product [56]. The availability of thorium sources provides the opportunity for cost savings related to target reprocessing procedures. Moreover, some of the current accelerators are well-equipped with the necessary infrastructure for target production, bombardment, and processing.

The major challenge facing this production method is the generation of additional isotopes other than [^225^Ac]Ac. In this case, post-irradiation separation processes will be required to recover the [^225^Ac]Ac radionuclide, which significantly elevates the production cost. For instance, the generation of ^227^Ac at the end of bombardment with an activity percentage of 0.1–0.2% strongly limits the use of [^225^Ac]Ac for clinical requirements. An alternative method for producing [^225^Ac]Ac is the ^232^Th(p,x)^225^Ac reaction, which utilizes high-energy protons (>100 MeV). This technique represents a promising avenue for exploring production options beyond the traditional [^229^Th]Th/[^225^Ra]Ra/[^225^Ac]Ac generator method. While only a limited number of facilities currently offer proton beams at the energy levels required for this nuclear reaction, this presents an opportunity to invest in and develop more facilities capable of producing these high-energy beams. Furthermore, the challenges of isolating [^225^Ac]Ac from the bulk thorium target and the various co-produced spallation products can be addressed through advancements in chemical separation techniques. It is important to consider the co-production of long-lived actinium-227 (^227^Ac), which has a half-life of 21.77 years. By focusing on these aspects, we can enhance the efficiency and feasibility of this production method [52]. To overcome this problem, most facilities tend to modify the current target processing method [57] by separating radium from the irradiated thorium a few days after the end of bombardment (EOB) to build a [^225^Ra]Ra/[^225^Ac]Ac generator. Some radium isotopes are also produced, such as [^228^Ra]Ra, [^226^Ra]Ra, [^225^Ra]Ra, [^224^Ra]Ra, and [^223^Ra]Ra, with half-lives of 5.7 y, 1600 y, 14.9 d, 3.6 d, and 11.4 d, respectively. Only [^228^Ra]Ra and [^225^Ra]Ra decay to actinium isotopes. Hence, using this combination as a radium/actinium generator can provide [^225^Ac]Ac free from toxic [^227^Ac]Ac. [^228^Ac]Ac (t_1/2_ = 6.2 h) will appear with an activity percentage of 0.88% after the separation of ^225^Ra/[^225^Ac]Ac. After an optimal [^225^Ac]Ac elution, [^228^Ac]Ac would normally decline with time [57]. [^228^Ac]Ac decays to [^228^Th]Th, from which [^225^Ac]Ac can be separated with a minor amount of impurities.

##### 3.2.3. [^225^Ac]Ac Production via Special Facilities

The promising therapeutic applications of [^225^Ac]Ac have a significant impact on establishing several nuclear facilities to overcome its limited availability. In 2000, the Isotope Separator and Accelerator Facility (ISAC) in TRIUMF, Canada, was commissioned and used to produce radioisotopes for specific research purposes. This facility is based on the bombardment of thorium or uranium targets with protons (480 MeV) to produce radioisotopes that can be extracted in a heterogeneous ion beam. These isotopes are then separated according to their mass by Isotope Separation On-Line (ISOL) techniques, producing a consistent isobaric ion beam [58]. [^225^Ra]Ra and [^225^Ac]Ac isotopes are separated by isolating the mass of 225, which are then implanted onto an aluminum target, followed by their extraction on a solid-phase resin that acts as a [^225^Ra]Ra/[^225^Ac]Ac generator and provides [^225^Ac]Ac by the decay of the parent [^225^Ra]Ra [59]. [^225^Ac]Ac production by ISAC has covered TRIUMF’s needs for [^225^Ac]Ac for radiolabeling investigations and preclinical studies. In 2016, the ISAC facility provided 44.4 MBq (~1.2 mCi), which can be theoretically scaled up to 370 MBq (~10 mCi) of [^225^Ac]Ac monthly.

TRIUMF also launched an isotope production facility (IPF) for [^225^Ac]Ac production targets by proton (500 MeV, 120 µA) bombardment of thorium targets using its primary beamline (BL1A). In 2017, IPF produced 370 MBq (~10 mCi) of [^225^Ac]Ac, reaching 3700 MBq (~100 mCi) by 2018 [45].

The ISOL technique to produce [^225^Ac]Ac has also been applied at ISOLDE at the European Organization for Nuclear Research (CERN) in Geneva, Switzerland. The ISOL technique provides pure [^225^Ac]Ac free from other actinium isotopes. However, the production yields remain unsatisfactory compared to the quantities produced by [^229^Th]Th generators. In 2017, CERN also started a new radioisotope production facility called MEDICIS, which produces mass-separated radioisotope beams using offline targets, planning to use uranium targets for [^225^Ac]Ac production. The expected production capacity of MEDICIS could yield 112 MBq (3 mCi) of [^225^Ac]Ac monthly.

##### 3.2.4. Transformation of [^226^Ra]Ra to [^229^Th]Th via Thermal Neutron Irradiation

Establishing alternative routes to increase [^229^Th]Th hoards would subsequently resolve the [^225^Ac]Ac supply shortage. This can be achieved by the irradiation of [^226^Ra]Ra targets with intense neutron fluxes in a reactor according to the following neutron capture reactions.

^226^ Ra(n,γ)^227^Ra

^227^ Ac(n,γ)^228^Ac

^228^ Th(n,γ)^229^Th

These reaction pathways have been tested in the High-Flux Isotope Reactor (HFIR) at ORNL, focusing on required target development and anticipated production outcomes [60]. The resulting amount of [^229^Th]Th produced is ~2.8 MBq per one gram of radium in a single day of irradiation [61]. Nevertheless, this procedure generates long-lived ^228^Th (t_1/2_ = 1.9116 a), which can be very difficult to separate from [^229^Th]Th. Moreover, as stated before in Section Challenges Encountered in the Use of Radium Targets, the use of [^226^Ra]Ra targets results in the evolution of the gaseous ^222^Rn daughter, causing serious difficulties with handling and managing radium targets [21,62]. All of these described production methods are briefly listed in Table 1.

## 4. [^225^Ac]Ac/[^213^Bi]Bi Radionuclide Generator

**Figure 2:**
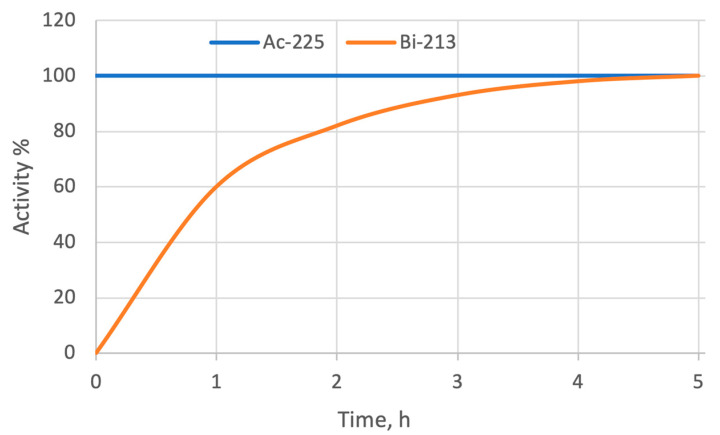
Growth of [^213^Bi]Bi activity from [^225^Ac]Ac decay after generator elution.

[^225^Ac]Ac/[^213^Bi]Bi radionuclide generator is the leading source for providing short-lived [^213^Bi]Bi [52]. Because of the half-life of the parent, [^225^Ac]Ac (t_1/2_ = 9.9 d), the working lifetime of the [^225^Ac]Ac/[^213^Bi]Bi generator is estimated to be several weeks. The generated daughter, [^213^Bi]Bi (t_1/2_ = 45.6 min), is known for its high specific activity regardless of the negligible percentage of the long-lived ^209^Bi (t_1/2_ = 2.01 × 10^19^ y) that may be present in the [^213^Bi]Bi eluate. Generally, according to the level of radioactivity needed per patient dose, [^225^Ac]Ac/[^213^Bi]Bi generators are usually eluted every two to three hours in medical practice, as can be seen in Figure 2, showing that [^213^Bi]Bi yield can reach 83% to 93% [52].

Many [^225^Ac]Ac/[^213^Bi]Bi generators have been developed depending on extraction chromatography or cation and anion exchangers [44,49,63,64,65,66,67,68,69]. Among these generators, [^225^Ac]Ac/[^213^Bi]Bi generators that are based on AG MP-50 cation exchange resin are the most commonly used systems [27,44,68,70], and they have been used in all [^213^Bi]Bi patient studies [30]. The Joint Research Centre (JRC) of the European Commission in Karlsruhe, Germany, has improved a high activity generator system that enables an efficient generator operation, especially when loaded with 4 GBq of [^225^Ac]Ac [28,71]. A crucial criterion of this generator system is the uniform activity distribution along nearly two-thirds of the generator resin to reduce the probable radiolysis of the organic resin and to ensure stable operation over several weeks. This generator has been effectively applied to prepare clinical doses of ~2.3 GBq [^213^Bi]Bi-Substance P analog for locoregional therapy for brain cancers [28].

An inverse generator protocol has also been proposed for the development of [^225^Ac]Ac/[^213^Bi]Bi generators. It is recommended because of its high radiation stability. In this method, the [^213^Bi]Bi that is generated is sorbed from [^225^Ac]Ac solution. Then, it is recovered for use in the intended application. Moreover, the design of these generators limits the co-production of ^227^Ac decay by-products. Based on this approach, the Pacific Northwest National Laboratory (PNNL) in the USA has improved multi-column selective inversion generators (MSID) [68]. This MSIG acts by the sorption of [^213^Bi]Bi on an anion exchanger, followed by its desorption from the column using sodium acetate buffer solution. Bray et al. developed a protocol for generator automization using sequential flow injection [66]. The main drawback of this procedure is the high percentage of [^225^Ac]Ac impurity in the product, which is ~ 0.1% of the total [^225^Ac]Ac activity. Furthermore, 2–3% of the parent [^225^Ac]Ac activity comes with each elution.

Betenekovet et al. proposed using annealed hydroxide inorganic sorbents with high radiation resistance [72]. These inorganic materials have shown high selective sorption affinity for [^213^Bi]Bi while [^225^Ac]Ac remains in the solution, favoring their use in the inverse generator design [72]. As a result, a novel [^225^Ac]Ac/[^213^Bi]Bi inverse generator was proposed and investigated, using a modified inorganic sorbent material consisting of zirconium and yttrium oxide mixtures that are thermally treated at 950 °C. The sorption of [^213^Bi]Bi was performed using a 0.1 M HNO_3_ solution, while the separation of [^213^Bi]Bi can be achieved using 1 M HCl. The optimal yield of [^213^Bi]Bi reached 98% using a 200 mg column, where almost 90% of [^213^Bi]Bi activity is concentrated in the first 0.3 mL of the eluate. Additionally, increasing the initial salinity of the [^225^Ac]Ac solution up to 3 M has increased the product purity to reach 0.01% of its activity.

### Quality Control Evaluation of Produced [^213^Bi]Bi

Due to the short half-life of [^213^Bi]Bi, its quality control evaluation is preferably carried out directly after elution to prevent radioactivity loss. The radiochemical purity of labeled [^213^Bi]Bi antibodies is usually performed using ITLC within 5 min, whereas [^213^Bi]Bi labeled peptides can be examined using ITLC or Radio-HPLC.

Conversely, owing to the low activity ratio of [^225^Ac]Ac to [^213^Bi]Bi (~10^−6^) and the reduced emission probability of gamma photons (˂2%) from [^225^Ac]Ac decay, quick and direct measurement of the parent [^225^Ac]Ac breakthrough is not applicable simultaneously with [^213^Bi]Bi. Hence, the measurement of [^225^Ac]Ac activity is performed after 24 h by gamma spectrometry, and, consequently, an appropriate determination of [^225^Ac]Ac radioactivity in the eluted [^213^Bi]Bi is generally assured indirectly using another generator of the same type. Labeled peptides and antibodies with [^213^Bi]Bi are allowed to contain less than 1 ppm [^225^Ac]Ac [73].

## 5. Radiolabeling Strategies for [^225^Ac]Ac and [^213^Bi]Bi

**Figure 3:**
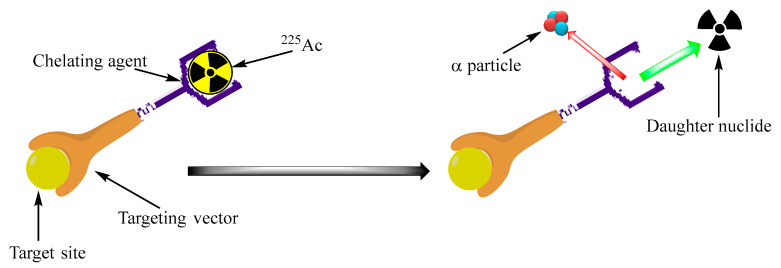
Instability of the daughter radionuclide/chelating agent complex as a result of [^225^Ac]Ac α-particle emission (recoil effect).

The emission of multiple alpha particles from [^225^Ac]Ac decay (Figure 1) makes it an effective radioisotope in cancer therapy. However, these emissions make the delivery of [^225^Ac]Ac and its daughter a huge challenge. Because of the conservation of momentum, the energetic alpha-particle emission imparts high recoil energy to the daughter nucleus (>100 KeV). This energy is 1000 times more than the binding energy of any chemical bond [74,75], resulting in the escape of the daughter nuclide from the delivery vector due to the chemical bonds rupturing (Figure 3) and increasing the toxicity of healthy tissue because of the next α or β emission. The recoil daughter ^221^Fr was accumulated mainly in the kidneys, while [^213^Bi]Bi was distributed in the kidneys, urine, and other organs by ratios of 40, 30, and 30%, respectively [76].

The main strategies for overcoming the recoil effect are summarized as follows.

### 5.1. Fast Uptake in Target Tissues

The rapid uptake of the radiopharmaceutical by the target tissue and its rapid excretion from the body will decrease the recoil phenomena. Antibodies and peptides are beneficial for highly specific cell targeting, but antibodies take a long time to reach the tumor site [77]. Using smaller targeting agents will help to increase the stability of these complexes. Many researchers performed many experiments to make [^225^Ac]Ac safer during its use in treatment [78,79,80]. [^225^Ac]Ac-DOTA-J591 was preclinically studied on prostate carcinoma and showed a high affinity to the cancer cell, which decreases the recoil phenomenon at a low dose of administered activity [80]. Many other studies have been evaluated using antibodies and peptides to decrease the presence of [^225^Ac]Ac daughter nuclides in the healthy tissues.

Nevertheless, in all cases, the renal toxicity must be decreased, which could be accomplished by using a metal chelate and a diuretic. For example, DMPS, 2,3-dimercapto-1-propanesulfonic acid, complexes with [^213^Bi]Bi and suppresses its uptake in the kidneys. Secondly, chlorothiazide and furosemide, as diuretics, inhibit [^221^Fr]Fr absorption in the renal tissue [81].

### 5.2. Encapsulation of the Radionuclide into a Nano-Carrier

It is expected that the encapsulation of alpha-emitting radionuclides into a nanoparticle cavity keeps the daughter nuclide retained inside the cavity or structure of the nano-carrier and only permits the release of alpha particles to perform their therapeutic effect. Nonetheless, there are many harmful effects of using nanoparticles for this purpose. One limitation of this therapeutic dynamic is the accumulation of nanoparticle-containing alpha-emitters inside the liver during the hepatobiliary/intestinal execratory pathway (due to its large size), which may cause hepatic damage [77]. Some of the literature illustrates strategies for the formation of a [^225^Ac]Ac-nanoparticle system [82,83,84,85,86,87]. Many attempts have been carried out to overcome the recoil effect using the nanotechnology therapeutic idea. Liposomes, for example, have been used in sizes between 100 and 800 nm, giving maximum [^213^Bi]Bi retention of only 12% [88]. Another study that used polymeric particles with sizes of ~800 nm succeeded in retaining 70% of [^213^Bi]Bi inside the carrier [86]. Almost all of the [^221^Fr]Fr and ~70% of [^213^Bi]Bi were retained in carriers by using multi-walled nanoparticles. The outer shell was comprised of gold, whereas the inner shell was gadolinium phosphate, and the conjugated core is [La_0.5_Gd_0.5_([^225^Ac]Ac)]PO_4_-mAb-201b. However, the large size of the nano-construct is still an obstacle to proper application. Smaller-sized nanoparticles may help to improve the excretion properties of recoiled daughter radionuclides from the body; however, this will also allow for the recoil daughters to escape in very high amounts [85].

### 5.3. Local Administration of the Alpha-Emitters

The direct injection of alpha-emitters into the target site helps to overcome the recoil effect of the alpha-emitted radionuclide [45] and a series of studies have been performed using this approach [89,90,91,92,93,94]. Substance P, for example, has been combined with [^213^Bi]Bi-DOTA complex and injected locally into gliomas in a clinical study [93].

## 6. Chelating Agents for [^225^Ac]Ac and [^213^Bi]Bi

**Figure 4:**
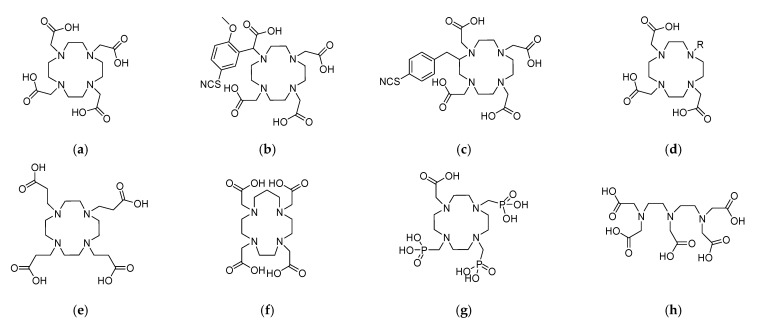

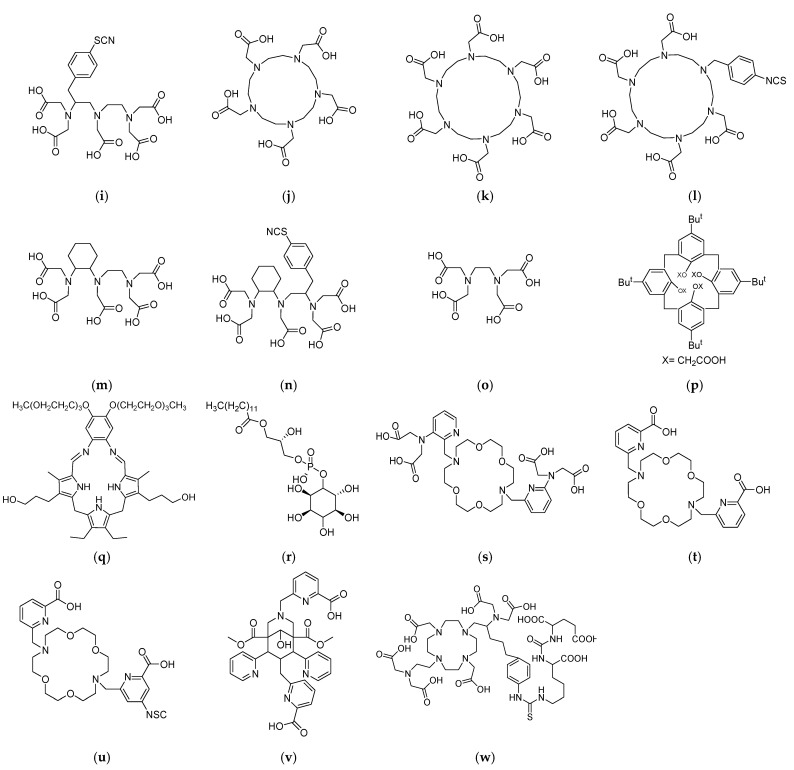
Chelating agents for [^225^Ac]Ac. (**a**) DOTA [38,80]. (**b**) MeO-DOTA-NCS [80]. (**c**) 2B-DOTA-NCS [80]. (**d**) DO3A [80]. (**e**) DOTPA [80]. (**f**) TETPA [80]. (**g**) DOTMP [80]. (**h**) DTPA [80]. (**i**) DTPA-p-Bn-NCS [80]. (**j**) PEPA [103]. (**k**) HEHA [104]. (**l**) HEHA-NCS [104]. (**m**) CHX-A′′-DTPA [103,105]. (**n**) CHX-A′′-DTPA-p-Bn-NCS [103,105]. (**o**) EDTA [105]. (**p**) t-Bucalix[4]arene-tetracarboxylic acid [24,27,106]. (**q**) Motexafin [24,107]. (**r**) L^py^ [24,107]. (**s**) Macropid [24,108]. (**t**) Macropa [24,108]. (**u**) Macropa-NCS [24,108]. (**v**) Bispa^2^ [24,108]. (**w**) EuK-106 [24,108].

The use of [^225^Ac]Ac as TAT requires not only consideration for the radiometal but also for the chelating agent and the targeting moiety. The development of a suitable chelating agent for [^225^Ac]Ac needs careful evaluation of the chemistry of [^225^Ac]Ac. However, there is a notable lack of understanding of its coordination chemistry as Ac^3+^. Its large ionic radius generates another challenge for the production of suitable metal complexing ligands. The unique chelating agents must offer fast coordination kinetics under mild conditions and also provide in vivo thermodynamic stability of the formed complex [49,62,63,64,95]. DOTA (1,4,7,10-tetraazacyclododecan-1,4,7,10-tetraacetic acid) and its derivatives are the most suitable chelating agents for [^225^Ac]Ac as of now. The optimum radiolabeling parameters include the addition of 0.02 M DOTA to NH_4_OAc at pH 6. Then, the mixture is incubated for 2 h at 37 °C, giving a very high radiochemical yield of 99% [45]. Studies have shown very high stability of the complex in preclinical studies with promising results [49,78,96,97,98,99,100,101,102]. Figure 4 summarizes the most widely used chelating agents for [^225^Ac]Ac.

[^213^Bi]Bi can be linked in a stable manner to biomolecules through diethylene triamine penta-acetic acid (DTPA) derivatives or 1,4,7,10-tetraazacyclododecan-1,4,7,10-tetraacetic acid (DOTA). These metal complexing agents are also most suitable for labeling [^213^Bi]Bi with antibodies because of their fast and stable complexation at ambient temperature [49,63,64,109,110,111]. Due to its high stability, the [^213^Bi]Bi-DOTA-complex has also been used for conjugation to peptides. The synthetic protocol for the preparation of [^213^Bi]Bi-DOTA complex provides radiochemical yields (RCY) ≥99% using microwave heating for less than 5 min at 95 °C (pH 8–9). Many preclinical and clinical studies have been performed using [^213^Bi]Bi as TAT (Table 2).

## 7. Biological Studies of [^225^Ac]Ac/[^213^Bi]Bi-Radiopharmaceuticals

Although α-emitters are considered promising and efficient radiotherapeutic agents, many application barriers restrict their market accessibility, especially their high toxicity [130]. The preclinical evaluation of α-emitters is mandatory, and many more in vitro and in vivo studies are necessary for routine clinical usage [131]. Generally, α-particles with 5–9 MeV emission energy show a mean path length of 40–100 µm and LET of ~80 keV/µm [130]. Thereby, they are considered promising radiotherapeutic candidates for metastatic castration-resistant prostate cancer, relapsed or refractory CD-22-positive non-Hodgkin lymphoma, acute myeloid leukemia, neuroendocrine tumors, ovarian carcinoma, gliomas, intralesional and systemic melanoma, colon cancer, and bone metastases [132]. A complete summary of the current progress of preclinical and clinical evaluations of [^225^Ac]Ac and [^213^Bi]Bi for the development of novel targeted alpha therapies for the treatment of different solid tumors is therefore necessary as a section of this body of work. Trials have been conducted to evaluate [^225^Ac]Ac/[^213^Bi]Bi radiopharmaceuticals on the preclinical scale, and some of these agents have been moved into phase 0, I, and II clinical investigations. For example, [^225^Ac]Ac-PSMA-617 was preclinically evaluated for prostate cancer therapy in PC-3/PC-3-PIP tumor-bearing mice [133]. Prostate-specific membrane antigen (PSMA) small molecules have also attracted attention in TAT, including PSMA-617 for prostate cancer therapy. The radio-conjugate [^225^Ac]Ac-PSMA-617 demonstrated improved therapeutic efficacy in PC-3/PC-3-PIP-tumor-bearing mice in comparison to [^177^Lu]Lu-PSMA-617 [133]. In addition, [^225^Ac]Ac-BC8 showed effective tumor control of tumors in a multiple myeloma tumor-bearing mouse model [134,135]. [^225^Ac]Ac-E4G10 has shown high efficacy for managing tumor growth in glioblastoma-bearing mice [136]. Moreover, [^225^Ac]Ac-PSMA-617 was clinically evaluated in patients with diffuse red marrow infiltration of mCRPC and peritoneal carcinomatosis with liver metastases. This radiopharmaceutical showed a drop in the PSA level for patients, confirming the effectiveness of the drug and the presence of treatment response [12].

### 7.1. Pre-Clinical Studies of [^225^Ac]Ac/[^213^Bi]Bi-Radiopharmaceuticals

Recent preclinical investigations of [^225^Ac]Ac/[^213^Bi]Bi-radiopharmaceuticals indicate the potential of being used as effective TAT agents [137]. A key criterion in designing TAT agents is the proper selection of a molecular platform that achieves the best radionuclide delivery to the target tissue as well as the availability of chemically active groups suitable for conjugation of the chelator. Targeting platform selection relies heavily on some critical factors that include off-target binding and its bio-pharmacokinetics behavior [138]. In addition to the traditional delivery platforms (small molecules, peptides, and antibodies), the new pipeline of nano-sized platforms, such as self-assembled vesicular structures and inorganic nanoparticles, has evolved, resulting in improved targeting ability, inducing high efficacy for a new therapeutic delivery platform [139].

#### 7.1.1. Traditional Delivery Platforms

Table 3 summarizes [^225^Ac]Ac/[^213^Bi]Bi conjugates that have undergone preclinical evaluation, demonstrating a therapeutic effect via the traditional delivery platforms that include peptides and antibodies.

##### Antibodies

Radio-immunotherapy (RIT) demonstrates the utilization of monoclonal antibodies (mAbs) that could be conjugated to chelating moieties in order to deliver radioactive payloads of particulate energy to cellular targets [138]. In vitro cytotoxicity investigations of [^225^Ac]Ac-DTPA-IgG1 and [^213^Bi]Bi-DTPA-IgG1 probes have been conducted on the human epidermoid A431 tumor cell line [156]. This study showed that the two probes have the ability to destroy tumor cells with a higher therapeutic propensity for the [^213^Bi]Bi as compared to the [^225^Ac]Ac probe. Another [^225^Ac]Ac-labeled antibody, [^225^Ac]Ac-DTPA-201B, has been investigated in vivo to target murine lung endothelial thrombomodulin [103]. Although the probe demonstrated potential lung targeting ability, it showed an unsatisfactory biological half-life in the target organ. Subsequently, another study utilized HEHA chelator for designing [^225^Ac]Ac-HEHA-201B to target lung carcinoma [142]. The in vivo distribution showed significant accumulation within the lung (300% ID/g) at 4 h post-injection and an improved biological half-life (49 h). However, regrettably, cleared [^225^Ac]Ac was accumulated primarily within the liver, spleen, and bone. Further studies have utilized CC49 antibody to formulate an [^225^Ac]Ac-HEHA-CC49 probe and its in vivo biodistribution has been investigated in a tumor-bearing mouse model (LS174T xenografts) post-subcutaneous (s.c) as well as -intramuscular (i.m.) delivery [152]. This demonstrated that the probe was accumulated in the tumor tissue with 25 and 8% ID/g at 24 h post s.c. and i.m. injection, respectively. Trastuzumab, an FDA-approved mAb for targeting HER2/*neu* in breast cancer malignancies, has been labeled with [^225^Ac]Ac, and its in vitro cytotoxicity was investigated against breast cancer spheroids MCF7, MDA-MB-361 (MDA), and BT-474 (BT) as a potential agent for RIT [157]. Recently, another study has been conducted on [^225^Ac]Ac-Trastuzumab for the effective treatment of ductal carcinoma [158]. Bioluminescent imaging post-intraductal administration of the drug revealed that [^225^Ac]Ac-Trastuzumab demonstrated better therapeutic efficacy in comparison to the intravenous route of administration by achieving systemic toxicity bypass. Another FDA-approved mAb, anti-VE-cadherin antibody (E4G10), was investigated as a potential TAT agent ([^225^Ac]Ac-E4G10) for targeting the vascular endothelium of glioblastoma with tumor growth-controlling ability in a mouse model [159]. Anti-CD45 antibodies have also been employed as targeting ligands for TAT [160]. Another study has utilized [^225^Ac]Ac-BC8 radio-conjugate as a potent targeting agent into organs of the immune system, especially bone marrow [161]. [^213^Bi]Bi radionuclide has been effectively used for TAT, where it was conjugated to the anti-CD33 antibody construct (HuM-195), establishing a [^213^Bi]Bi-HuM-195 radio-conjugate [162]. Another study has utilized 9E7.4 anti-CD138 antibodies as targeting moieties in TAT based on [^213^Bi]Bi [126]. The study revealed that [^213^Bi]Bi-9E7.4 anti-CD138 is more effective than [^177^Lu]Lu-9E7.4 anti-CD138 in mice bearing 5T33 multiple myeloma tumors, where there is a significant increase in the mice survival at 45%. A subsequent study utilized J591 anti-PSMA mAb for designing a TAT agent ([^213^Bi]Bi-J591) for the treatment of prostate cancer [163]. The study revealed that [^213^Bi]Bi-J591 is a potential cytotoxic agent against LNCaP spheroids in vitro and in vivo.

##### Peptides

Since octreotide, an analog of the endogenous peptide hormone somatostatin, was approved by the FDA, peptide receptor radionuclide therapy (PRRT) has gained much focus from many researchers around the globe [138]. However, PRRT agents are facing a heightened limitation in that they are compromised by nephrotoxicity [164]. The first reported literature about integrating octreotide analogs as targeting TAT moieties was demonstrated in 2006 [154]. This study revealed that the octreotide analog (DOTATOC), when radiolabeled with the high-LET α-emitter [^213^Bi]Bi, showed promise in in vivo distribution, toxicity, safety profile, and therapeutic efficacy in a rat pancreatic carcinoma model. The results demonstrated that [^213^Bi]Bi-DOTATOC radio-conjugate accumulated specifically in tissues rich with somatostatin receptors. It also showed dose-dependent antitumor efficacy in tumor reduction when rats were treated with >20 MBq of [^213^Bi]Bi-DOTATOC in comparison to the rodents receiving <11 MBq of conjugate. Interestingly, the study revealed little nephrotoxicity at dosing levels of radioactivity from 4 to 22 MBq. Subsequently, [^225^Ac]Ac-DOTATOC radio-conjugate was developed, and the in vivo distribution was investigated against pancreatic neuroendocrine tumors in a xenografted mouse model [78]. The results showed good accumulation of [^225^Ac]Ac-DOTATOC in neuroendocrine xenografted tumors with improved therapeutic efficacy at activity greater than 30 kBq of the drug. Recently, preclinical investigations have been conducted on [^213^Bi]Bi-DOTATATE to evaluate the therapeutic efficacy in athymic mice pre-treated with and without L-lysine as a renal protecting agent [165]. The results demonstrated a good tumor accumulation in both cases, while the renal uptake in the case of L-lysine pre-treated mice showed half the accumulation compared to the non-treated mice. The maximum tolerated dose (MTD) of [^213^Bi]Bi-DOTATATE increased from 13.0  ±  1.6 MBq in the non-treated mice to 21.7  ±  1.9 MBq in L-lysine pre-treated mice. An additional investigation has evaluated the therapeutic efficacy of [^213^Bi]Bi-DOTATATE against different-sized (small and large) tumor lesions utilizing two tumor models: H69 (human small cell lung carcinoma) and CA20948 (rat pancreatic tumor) [166]. The results revealed greater tumor accumulation of conjugate (19.6 ± 6.6% ID/g) in the case of the CA20948 model as compared to 9.8 ± 2.4% ID/g in the case of H69. Nevertheless, the antitumor efficacy of [^213^Bi]Bi-DOTATATE was greater in the case of the H69 tumor model, with a higher survival rate. Also, it revealed that no tumor regrowth was observed in the case of small tumor groups (H69 model) [165].

#### 7.1.2. Advanced Delivery Platforms

The application of nano-constructs as delivery platforms in TAT has received significant attention from researchers and clinicians around the world. It provides the possibility of overcoming the difficulties associated with traditional approaches, which include the recoil effect of the daughter radionuclide and the insufficiency of efficient chelating agents for complexing α-radionuclides to the molecular target [167]. Table 4 summarizes the preclinical studies of [^225^Ac]Ac/[^213^Bi]Bi-nanoradiopharmaceuticals.

##### Self-Assembled Vesicles

Liposomes

Multi-vesicular liposomes (MUVELs) have shown potential usefulness for the appropriate delivery of [^225^Ac]Ac radionuclide to ovarian cancer cells [75,88]. This study demonstrated the conjugation of MUVELs with HER2/neumAb (Trastuzumab) and evaluated the therapeutic efficacy in vitro against ovarian cancer cells. The results revealed an appropriate reduction in the recoil effect of the daughter [^213^Bi]Bi radionuclide (17–18% after 20 days). Also, biological investigations revealed high-affinity binding to ovarian cancer cells with improved cell uptake kinetics. Another study has demonstrated the usage of [^225^Ac]Ac-labelled liposomes for the treatment of prostate cancer [82]. The authors of this study demonstrated appropriate [^225^Ac]Ac radionuclide loading to J591 mAb and the A10 aptamers conjugated to a liposome construct to target the PSMA protein. The in vitro investigation revealed that [^225^Ac]Ac-J591-liposomes had exhibited a greater cytotoxic effect than [^225^Ac]Ac-A10 aptamers-liposomes, encouraging the significance of [^225^Ac]Ac-J591-liposomes as a PSMA-TAT agent. Another study conducted the utilization of [^213^Bi]Bi-radiolabeled immunoliposomes as a potential agent for the treatment of early-stage metastases [177]. [^213^Bi]Bi was conjugated to liposomal-CHX-A-DTPA nano-construct (100 nm), and the therapeutic efficacy was evaluated against a rat/*neu* transgenic mouse model of mammary carcinoma. The results revealed an increase in the survival time of 38 days as compared to 29 days for the [^213^Bi]Bi-CHX-A-DTPA radio-conjugate.

Polymersomes

Compared to liposomes, polymersomes (self-assembled polymeric amphiphiles) can demonstrate higher stability and reduced permeability in human tissue. Consequently, they have become a potential platform to reduce the recoil effect of the daughter radionuclides [171]. The first attempt to formulate polymersomes encapsulated with [^225^Ac]Ac radionuclide to reduce the recoiling effect demonstrated that double-layered polymersomes (400 nm) achieved 47% of [^213^Bi]Bi recoil reduction, while the single-layered derivative achieved only 10–20% [86]. Furthermore, it was postulated that increasing the polymersome size to 800 nm could reduce the [^213^Bi]Bi recoils by 80%. The therapeutic efficacy of [^225^Ac]Ac-labeled polymersomes has been investigated against U87 human glioma spheroids. An appropriate distribution of polymersome was achieved after only four days [169]. This study showed that 0.1 kBq [^225^Ac]Ac-labeled polymersome caused a reduction in spheroidal growth and that increasing the activity to 5 kBq caused complete tumor death after only two days. An additional investigation has compared the recoil effect of [^225^Ac]Ac-DTPA with [^225^Ac]Ac-doped-InPO_4_ immobilized polymersome (200 nm) [170]. The in vivo distribution post-intra-tumor (i.t.) injection in mice bearing MDA-MB-231 tumors revealed high activity accumulation of the polymersome in the tumor lesions, while [^225^Ac]Ac-DTPA was mainly accumulated and excreted rapidly through the renal–urinary excretion pathway. Recoil studies showed that [^213^Bi]Bi was retained in the tumor lesion in the case of the polymersome formulation, while more [^213^Bi]Bi was retained in the kidney in the case of [^225^Ac]Ac-DTPA complex. Studies conducted using sticky lipid vesicles with clustered HER2-targeting peptides loaded with [^225^Ac]Ac radionuclide for the treatment of breast cancer [175] have demonstrated that the sticky vesicles have a propensity to target HER2-negative breast cancer cells (MDA-MB-231 and MCF7) with some cytotoxic effect of 42–61%.

##### Carbon Nano-Construct

As an alternative delivery platform, carbon nanotubes (CNTs) for the immobilization of [^225^Ac]Ac radionuclide have been employed by Ruggiero et al. [178]. The study developed DOTA-CNTs functionalized with E4G10 antibody for targeting the tumor vascular endothelial-cadherin. The in vivo investigation of [^225^Ac]Ac- CNT-DOTA-E4G10 against mice induced with LS174T (colon) adenocarcinomas showed appropriate tumor reduction with a two-fold higher survival rate compared to controls. In another study involving CNTs for the targeted delivery of [^225^Ac]Ac radionuclide to the tumor, a pre-targeting approach was employed using mAb-MORF [174]. [^225^Ac]Ac-DOTA-CNT modified with mAb-MORF was developed, and in vitro investigations showed high-affinity binding to cancer cells. The in vivo evaluation in a lymphoma xenografted mouse model indicated improved therapeutic efficacy in a two-step approach, where complete tumor regression was achieved with rapid clearance.

##### Inorganic Nanoparticles

The utilization of lanthanum phosphate nanoparticles as a potential delivery platform for [^225^Ac]Ac radionuclide to target tumor lung vasculature with a high recoil effect reduction has provided some interesting results as well [75,172]. Here, the authors developed [^225^Ac]Ac-doped lanthanum phosphate nanoparticles functionalized with mAb 201B. In vivo studies for the i.v. administration of La-[^225^Ac]Ac-PO_4_ NPs-mAb201B in mice showed rapid and high accumulation within the lungs (30% of the total injected dose at 1 h post-injection). In addition, the authors declared that the lung had retained about 50% of the recoiled daughter radionuclides.

A study demonstrating the loading of [^225^Ac]Ac to gold-decorated lanthanide phosphate nanoparticles functionalized with mAb 201b antibody to produce [^225^Ac]Ac-(La_0.5_Gd_0.5_)PO_4_-mAb 201b aimed to reduce the recoil effect of the daughter radionuclides and to target tumor lung vasculature [85]. The in vivo distribution analysis of this new agent showed high accumulation within lungs, with 69% [^213^Bi]Bi retention in lung tissue with only 2.8% within the kidney at 1 h post-injection. Cedrowska et al. reported the demonstration of TiO_2_ nanoparticles functionalized with silane-PEG-SP(5-11) conjugates and labeled with [^225^Ac]Ac radionuclide to produce [^225^Ac]Ac-TiO_2_-silane-PEG-SP(5-11) as a potential TAT-nano-construct for brain tumors. This study showed appropriate cytotoxic action against glioblastoma cancer cells as revealed during in vitro investigations [173].

Gold nanoparticles are another inorganic delivery platform that has been used to design [^225^Ac]Ac-radiopharmaceuticals. Recently, Salvanou et al. have produced [^225^Ac]Ac-Au-TADOTAGA for localized cancer treatment [176]. This in vitro investigation against U87 glioblastoma cancer cells showed that [^225^Ac]Ac-Au-TADOTAGA demonstrated a cytotoxic effect with more than 70% cell killing after 48 h. Additionally, in vivo investigation revealed high accumulation (60.67 ± 3.87% ID/g) of drug within the tumor lesion at 2 h post i.t. injection, which decreased slowly over time.

### 7.2. Clinical Trials of [^225^Ac]Ac/[^213^Bi]Bi-Radiopharmaceuticals

Table 5 summarizes most of the [^225^Ac]Ac/[^213^Bi]Bi-radiopharmaceuticals that have been evaluated preclinically or under clinical phase investigations [11,14,22,36,106,109,114,121,122,123,133,134,135,136,179,180,181,182,183,184,185,186,187,188,189,190,191,192,193,194]. Clinical trial phase investigations for [^225^Ac]Ac/[^213^Bi]Bi-radiopharmaceuticals have begun and have been evaluated in ~1000 patients suffering from many different tumors. These studies have been largely based upon radiolabeling of cell-targeting antibodies, although other platforms, as described herein, have also taken precedence. Examples of several of these agents are listed below.

1. [^225^Ac]Ac-PSMA -617 was evaluated clinically in ~500 prostate tumor patients. It was used with an average dose of 100 kBq/kg body weight. It showed excellent radiotherapeutic action with an extensive decrease in prostate-specific antigen (PSA) [121,187,188,191,193,195,196].
2. [^213^Bi]Bi-anti-CD33-mAb was therapeutically efficient in clinical trials in ~150 leukemia patients. It reached phase I and II stages, and showed a safe dose of up to 3 µCi/kg with no acute toxicity effects [106].
3. [^213^Bi]Bi- Substance P has been used in ~100 glioma patients, resulting in promising extensive radiotherapeutic action with a dose of about 2 GBq in 2-month intervals [93,190,194,197].
4. [^213^Bi]Bi-anti-MCSP-mAb was investigated in ~65 melanoma patients to evaluate its ability to deliver an appropriate radiation load to the tumor. However, its pharmacokinetic behavior failed to reach the required therapeutic dose [122,123].
5. [^213^Bi]Bi-DOTATOC was tested in ~50 neuroendocrine tumor patients to evaluate its toxicity. It showed safe usage with radioactivities of 18.5 MBq in two-month dosing intervals. However, hepatotoxicity was a limiting parameter for use [14].
6. [^225^Ac]Ac/[^213^Bi]Bi-anti-EGFR-mAb was used via the intra-vesical route of administration in ~12 bladder cancer patients. It extensively reduced the toxicity to neighboring tissues with excellent therapeutic activity at the site of application [109].

## 8. Conclusions

This article underlines the potential role of alpha emitters, especially [^225^Ac]Ac and [^213^Bi]Bi radionuclides, as potential leaders for future TAT. Undoubtedly, because of their short penetration length in human tissues and their high LET, alpha emitters offer a therapeutic opportunity for patients who do not respond to conventional therapies. Preliminary studies have shown the advantageous utilization of [^225^Ac]Ac and [^213^Bi]Bi radiopharmaceuticals for cancer therapy. However, some challenges still remain and need to be carefully explored and resolved. For example, new and competitive production techniques have to be designed and tested to provide a sufficient supply of both radionuclides. In addition, their chemical and coordination behavior still requires resolution via extensive research considerations. Moreover, the scientific community must conduct additional clinical and preclinical studies on a larger scale in order to provide for more reliable and consistent results for using [^225^Ac]Ac and [^213^Bi]Bi labeled radiopharmaceuticals if routine usage for cancer treatments is to become relevant. Clinical applications of [^225^Ac]Ac/[^213^Bi]Bi-radiopharmaceuticals are still in the early phases of evaluation but still face many challenging issues that scientists are working intensively to resolve. The success of solving these complex research problems and achieving these goals will support TAT, introducing high LET radiation to tumor cells, producing a high payload of cytotoxic effect on them. Lastly, it is worth mentioning that the use of [^225^Ac]Ac/[^213^Bi]Bi-radiopharmaceuticals is still in its infancy, which means that growth is needed in terms of the production capacity and clinical application scales necessary for success. The scientific community has overcome and achieved many of these milestones in recent years. However, undoubtedly, there is still room to continue seeking a high level of research effort. It is pertinent to point out that continuous research cooperation on a global scale can exponentially increase the growth of using [^225^Ac]Ac/[^213^Bi]Bi-radiopharmaceuticals as influential TAT leaders in the very near future.

## Figures and Tables

**Table 1:** Current and prospective production approaches for [^225^Ac]Ac.

[^225^Ac]Ac Production		Production Mode	Production Facility	Refs.
Current	[^229^Th]Th	[^229^Th]Th generators: Natural decay of ^233^U hoards	ORNL, USA I.T.U., Germany IPPE, Russia	[27,28,31,32,33,35]
Developed	[^226^Ac]Ac	Electron Accelerators: ^226^Ra(γ,n)^225^Ra → ^225^Ac	Medical linacs: ARIEL (TRIUMF, Canada)	[39,43]
		Proton Accelerators ^226^Ra(p,2n) ^225^Ac	Medium-sized cyclotrons: JRC cyclotron, Germany ^1^ (QST), Japan ^2^ NPI, Czech Republic ^3^ CNEA, Argentina ^4^ SCK-CEN, Belgium ^5^ KIRAMS, South Korea	[21,40,41,46]
		Nuclear Reactors: ^226^Ra(n,2n)^225^Ra → ^225^Ac	Fast breeder reactor	[50]
	**[**^232^Th]Th	Thorium Spallation:^232^Th(p,x) ^225^Ac	INR, Russia BNL, USA LANL, New Mexico	[31,32,35,41,42]
		Special facilities	ISOL, ISAC (TRIUMF, Canada) ISOL, ISOLDE (CERN, Geneva) MEDICIS (CERN, Switzerland) IPF (TRIUMF, Canada)	[46,58,59]
	Transformation of [^226^Ra]Ra to [^229^Th]Th	^226^Ra(n,γ)^227^Ra,^227^Ac(n,γ)^228^Ac, ^228^Th(n,γ) ^229^Th	High-Flux Isotope Reactor (HFIR), ORNL, USA	[60,61]

^1^ (QST.): National Institutes for Quantum and Radiological Sciences and Technology; ^2^ (NPI): Nuclear Physics Institute; ^3^ (CNEA): Argentine National Atomic Energy Commission; ^4^ (CK-CEN): Nuclear Energy Research Centre; ^5^ (KIRAMS): Korean Institute for Radiological and Medical Sciences.

**Table 2:** An overview of the most-used chelating agents for [^225^Ac]Ac/[^213^Bi]Bi-labeled molecules.

Targeting Agent	Chelating Agent	Refs.
Anti-CD33 IgG (HuM195)	DMSA, DMPS, Ca-DTPA, SCNCHX-A-DTPA	[106,112,113]
Anti-CD20 IgG (rituximab)	DMPS, CHX-A′′-DTPA	[114,115]
Plasminogen activator inhibitor type 2	CHX-A′′-DTPA, cDTPA	[116,117,118]
Anti-MUC1 IgG (C595 IgG)	cDTPA, CHX.A′′	[117,119]
Substance P	DOTAGA-/DOTA	[93,120]
Anti-NG2 IgG (9.2.27 IgG)	cDTPA	[121,122,123,124,125]
Anti-CD138 IgG	p-SCN-Bn-DOTA, SCN-CHX-A′′-DTPA	[126]
Anti-PSMA IgG (J591 IgG)	cDTPAa	[127]
C6.5K-A scFv, C6.5K-A diabody	CHX-A′′-DTPA	[128]
Anti-EGFR-mAb	p-SCN-Bn-CHX-A′′-DTPA, CHX-A′′-DTPA	[109,129]
DOTATOC	DOTA	[14]

**Table 3:** [^225^Ac]Ac/[^213^Bi]Bi conjugates in preclinical evaluation that demonstrate the traditional delivery platforms.

Isotope	Compound	Targeting Moiety	Cancer Type	Result	Ref.
[^225^Ac]Ac	[^225^Ac]Ac–DOTA–HuM195	HuM195 Ab	Cynomolgus monkey leukemia.	Improved efficacy with less renal toxicity.	[100]
	[^225^Ac]Ac–DOTA-trastuzumab	Trastuzumab	SKOV3 human ovarian cancer.	Improved efficacy with less toxicity.	[68]
	[^225^Ac]Ac–DOTA–J591	J591 mAbs	Human LNCaP prostate.	Improved efficacy with no toxicity.	[70]
	[^225^Ac]Ac–DOTA–3F8	3F8 Ab	NMB-7 human neuroblastoma xenografts in nude mice.	Increased survival with low toxicity.	[140]
	[^225^Ac]Ac-7.16.4	7.16.4 mAb	neu-N transgenic mouse model with rat HER-2/neu expression and spontaneous lung metastases.	Improved efficacy with slight renal toxicity.	[141]
	[^225^Ac]Ac–DOTA–E4G10	E4G10 Ab	LS174T human colon xenografts in female nude mice.	High affinity targeting with appropriate efficacy.	[96]
	[^225^Ac]Ac-HEHA-Mab 201B	Mab 201B	EMT-6 mammary carcinoma.	Appropriate efficacy with some toxicity.	[142]
	[^225^Ac]Ac-DOTATOC	DOTATOC peptide	AR42J rat pancreatic exocrine.	Appropriate efficacy with less toxicity.	[78]
	[^225^Ac]Ac–DOTA–F3	F3 peptide	MDA-MB-435 human peritoneal carcinomatosis in SCID mice.	Improved targeting and efficacy with minor renal toxicity.	[79]
	[^225^Ac]Ac-Pep-1L	Pep-1L peptide	U8251 human glioblastoma orthotopic xenografts in male nude mice.	U8251 human glioblastoma. Orthotopic xenografts in male nude mice.	[143]
	[^225^Ac]Ac-DOTA–MC1RL	MC1RL peptide	MEL270 human uveal melanoma xenografts in SCID mice.	Decreased metastasis and improved survival.	[144]
	[^225^Ac]Ac-DOTAZOL	Zoledronic acid	Wistar rats.	Improved bone/blood ratio with some renal toxicity.	[145]
[^213^Bi]Bi	[^213^Bi]Bi-30F11-CHX-A	30F11 Ab	Female BALB/c mice.	High renal toxicity.	[146]
	[^213^Bi]Bi-CD138	Anti-CD138 Ab	5T33 mouse multiple myeloma cell culturedinC57BL/KaLwRij mice.	Improved survival with moderate toxicity.	[147]
	[^213^Bi]Bi-d9Mab	d9Mab	HSC45-M2 human gastric cancer cells in nude mice.	Improved efficacy with low toxicity.	[148]
	[^213^Bi]Bi-matuzumab	Matuzumab	EJ28 human orthotopic bladder xenografts in nude mice.	Increased efficacy with some renal toxicity.	[129]
	[^213^Bi]Bi-Fab	CO17-1A Fab	GW-39 human colon cancer in xenograft mice.	High efficacy with low toxicity.	[149]
	[^213^Bi]Bi-CHX-A-DTPA HuM195	HuM195 mAb	Normal BALB/c mice.	Improved pharmacokinetic parameters.	[150]
	[^213^Bi]Bi-d9-E-cad mAb	d9-E-cad mAb	HSC45-M2 human gastric xenografts with d9-E-cad mutation in female nude mice.	Improved efficacy upon double administration compared to a single one, with no toxicity.	[151]
	[^213^Bi]Bi- HuCC49DCH2	Humanized CC49 mAb	LS-174T human colon xenografts in female nude mice.	Improved efficacy.	[152]
	[^213^Bi]Bi-P-P4D	P-P4D peptide	OV-MZ-6 human ovarian xenografts in female nude mice.	High tumor accumulation with low nephrotoxicity.	[153]
	[^213^Bi]Bi-DOTATOC	DOTATOC peptide	CA20948 rat pancreatic adenocarcinoma tumors in Lewis rats.	Improved efficacy with low toxicity.	[154]
	[^213^Bi]Bi–DOTA–PESIN, or [^213^Bi]Bi-AMBA	PESIN and AMBA Peptides	PC-3 human prostate xenografts in female nude mice.	[^213^Bi]Bi–DOTA–PESIN had lower nephrotoxicity than [^213^Bi]Bi-AMBA.	[155]

**Table 4:** Summary of the preclinical studies of [^225^Ac]Ac/[^213^Bi]Bi-Nanoradiopharmaceuticals.

Isotope	Nano-Platform	Targeting Moiety	Cancer Type	Objective	Ref.
[^225^Ac]Ac	PEGylated liposome	PSMA J591 antibody	(PSMA)- expressing cells	Reduction in the recoil effect	[82]
	PEGylated liposome	Trastuzumab	SKOV-3 ovarian cells	Recoiling	[168]
	PEGylated liposome	-	-	Recoiling	[88]
	PEGylatedMUVEL	Trastuzumab	SKOV-3 ovarian cells	Recoiling	[81]
	Polymersomes	-	-	Recoiling	[169]
	Polymersomes	-	-	Recoiling	[170]
	Polymersomes	-	-	Recoiling	[171]
	(La_0.5_Gd_0.5_)PO_4_	MAb 201b	EMT-6 lung tumor cells	Recoiling	[85]
	La ([^225^Ac]Ac)PO_4_	MAb 201b	EMT-6 lung tumor cells	Recoiling	[172]
	TiO_2_	Substance P (5-11)	NK1 glioma receptor	Recoiling	[173]
	Carbon nanotubes-DOTA	LintuzumabRituximabanti-A33	CD20 + B-cell lymphoma C33 + myelocytic leukemia A33 + colon adenocarcinoma	Targeted delivery and rapid clearance	[174]
	Lipid vehicle	Trastuzumab	BT-474, MDA-MB-231, MCF7 breast carcinoma cell	Improved targeting of low HER2-expression cells	[175]
	Carbon nanotubes-DOTA	Tumor neovascular-targeting antibody	LS174T xeno-graft tumor model	Targeted delivery and rapid clearance	[136]
	Gold NPs-DOTA	-	U87 glioblastoma cancer cells	Localized cancer treatment	[176]
[^213^Bi]Bi	Immunoliposomes	CHX-A-DTPA	Rat/*neu* transgenic mouse model of mammary carcinoma	Improve the therapeutic efficacy	[177]

**Table 5:** [^225^Ac]Ac/[^213^Bi]Bi-radiopharmaceuticals on preclinical and clinical phases.

Tumor	[^225^Ac]Ac Agent	[^213^Bi]Bi Agent
Neuroendocrine	[^225^Ac]Ac-DOTATOC	[^213^Bi]Bi-DOTATOC
Multiple myeloma	[^225^Ac]Ac-BC8	[^213^Bi]Bi-labeled 9.E7.4 anti-CD138 mAb
	[^225^Ac]Ac-lintuzumab	[^213^Bi]Bi-labeled Anti-CD138 IgG
Metastatic castration resistant prostate cancer	[^225^Ac]Ac-PSMA617	[^213^Bi]Bi-DOTA-PESIN
Glioblastoma	[^225^Ac]Ac-E4G10	[^213^Bi]Bi-DOTA-substance P
Lymphoma	[^225^Ac]Ac-anti-CD33 HUM195	[^213^Bi]Bi-DOTA-biotin
		[^213^Bi]Bi-anti-CD20-mAb 12
Leukemia	[^225^Ac]Ac-lintuzumab	[^213^Bi]Bi-lintuzumab
	[^225^Ac]Ac-anti-CD33-mAb	[^213^Bi]Bi-HuM195
		[^213^Bi]Bi-anti-CD33-mAb 49
Breast cancer	[^225^Ac]Ac-7.16.4 anti-rat HER-2/neu	[^213^Bi]Bi-CHX-A″DTPA-C6.5K-A scFv
		[^213^Bi]Bi-Plasminogen activator inhibitor type 2
Melanoma	[^225^Ac]Ac-crown-_MSH	[^213^Bi]Bi-anti-MCSP-mAb 54
		[^213^Bi]Bi-Cdtpa-Anti-NG2 IgG (9.2.27 IgG)
Glioma	[^225^Ac]Ac-Substance P 20	[^213^Bi]Bi-Substance P 68
Neuroendocrine Tumors	[^225^Ac]Ac-DOTATOC 39	[^213^Bi]Bi-DOTATOC 25
Prostate cancer	[^225^Ac]Ac-PSMA617 > 400	[^213^Bi]Bi- cDTPAa- Anti-PSMA IgG (J591 IgG)
Advanced refractory Solid tumors	[^225^Ac]Ac-FPI-1434	Information is not available
Colorectal cancer	Information is not available	[^213^Bi]Bi-labeled CO-1A Fab’
Bladder carcinoma	Information is not available	[^213^Bi]Bi-anti-EGFR mAb
Peritoneal carcinoma	Information is not available	[^213^Bi]Bi-d9MAb
Ovarian cancer	Information is not available	[^213^Bi]Bi-CHX-A″DTPA-C6.5K-A scFv
		[^213^Bi]Bi-CHX-A″DTPA- C6.5K-A diabody
		[^213^Bi]Bi-Anti-MUC1 IgG (C595 IgG)
Non-Hodgkin lymphoma	Information is not available	[^213^Bi]Bi-Anti-CD20 IgG (rituximab)
Pancreatic cancer	Information is not available	[^213^Bi]Bi-Plasminogen activator inhibitor type 2
		[^213^Bi]Bi-Anti-MUC1 IgG (C595 IgG)

## Data Availability

The original contributions presented in this study are included in the article. Further inquiries can be directed to the corresponding author.

## Abbreviations

Ac	Actinium
Po	Polonium
Tl	Thallium
Rn	Radon
U	Uranium
Bq	Becquerel
keV	Kilo electron volt
Y	year
H	hour
A	alpha
t_1/2_	Half-life
W	Watt
N	Neuton
CK-CEN	Nuclear- Energy Research Centre
IPF	Isotope production facility
KIRAMS	Korean Institute for Radiological and Medical Sciences
CNEA	Argentine National Atomic Energy Commission
MSIG	multicolumn selective inversion generators
INR	Institute for Nuclear Research of the Russian Academy of Sciences
BNL	Brookhaven National Laboratory
linac	linear electron accelerator
ITU	Institute for Trans-Uranium Elements
ISOL	Isotope Separation On-Line
SRS	sealed radioactive sources
USA	United States of America
ITLC	Instant thin-layer chromatography
SPECT	Single-photon emission computed tomography
TAT	Targeted alpha-particle therapy
LET	Linear energy transfer
DTPA	Diethylenetriaminepenta acetic acid
DMPS	2,3-Dimercapto-1-propanesulfonic acid
PEPA	1,4,7,10,13-pentaazacyclopentadecane-N,N′,N′′,N′′′,N′′′′-pentaacetic acid
DOTPA	1,4,7,10-tetraazacyclododecan-1,4,7,10-tetrapropionic acid
p-SCN-Bn-DOTA	S-2-(4-Isothiocyanatobenzyl)-1,4,7,10-tetraazacyclododecane tetraacetic acid
DTPA-p-Bn-NCS	S-2-(4-Isothiocyanatobenzyl)-diethylenetriamine pentaacetic acid
2B-DOTA-NCS	2-(p-isothiocyanatobenzyl)-1,4,7,10-tetraazacyclododecane-1,4,7,10-tetraacetic acid
SCN-CHX-A-DTPA	2-(4-isothiocyanatobenzyl)diethylenetriamine pentaacetic acid
CHX-A″-DTPA	A′′ isomer of N-[(R)-2-amino-3-(4-nitrophenyl)propyl]-trans-(S,S-cyclohexane-1,2-diamine-N,N,N′,N′′,N′′-pentaacetic acid
MeO-DOTA-NCS	α-(5-isothiocyanato-2-methoxyphenyl)-1,4,7,10-tetraazacyclododecane-1,4,7,10-tetraacetic acid
HEHA-NCS	2-(4-isothiocyanatobenzyl)-1,4,7,10,13, 16-hexaazacyclohexadecane- 1,4,7,10,13,16-hexaacetic acid
Bispa^2^	(6,6′-((9- hydroxy-1,5-bis(methoxycarbonyl)-2,4-di(pyridin-2-yl)-3,7-diazabicyclo [3.3.1]nonane3,7-diyl)bis(methylene))dipicolinic acid)
SCN-CHX-A′′-DTPA or CHX-A′′-DTPA-p-Bn-NCS	[(R)-2-Amino-3-(4-isothiocyanatophenyl)propyl]-trans-(S,S)-cyclohexane1,2-diamine-pentaacetic acid
Bi	Bismuth
Fr	Francium
Pb	Lead
Ra	Radium
NPI	Nuclear Physics Institute
Ci	Curie
MeV	Mega electron volt
d	Day
s	Second
γ	Gamma
β	Beta
ppm	Part per million
p	Proton
JRC	Joint Research Centre
PNNL	Pacific Northwest National Laboratory
ISAC	Isotope Separator and Accelerator Facility
CERN	European Organization for Nuclear Research
QST	National Institutes for Quantum and Radiological Sciences and Technology
IPPE	Lipinski Institute for Physics and Power Engineering
LANL	Los Alamos National Lab
ARIEL	Advanced Rare IsotopE Laboratory
ORNL	Oak Ridge National Laboratory
IAEA	International Atomic Energy Agency
HFIR	High-Flux Isotope Reactor
EOB	end of bombardment
HPLC	High-pressure liquid chromatography
PC	Paper chromatography
RCY	Radiochemical-yield
EDTA	Ethylenediaminetetra acetic acid
DMSA	Dimercapto succinic acid
DOTA	(1,4,7,10-tetraazacyclododecan-1,4,7,10-tetraacetic acid)
DO3A	1,4,7,10-Tetraazacyclododecane-1,4,7-triacetic acid
cDTPA	Cyclic diethylenetriaminepenta acetic acid
TETPA	1,4,8,11-tetraazacyclotetradecan-1,4,8,11-tetrapropionic acid
DOTATOC	1,4,7,10-tetra-azacylododecane N,N′,N′′,N′′′-J-tetraacetic acid-Tyr3-octreotide
DOTMP	(1,4,7,10-tetraazacyclododecan-1,4,7,10-tetramethylene-phosphonic acid)
DOTAGA	2,2′,2′′-(10-(2,6-dioxotetrahydro-2H-pyran-3-yl)-1,4,7,10-tetraazacyclododecane-1,4,7-triyl)triacetic acid
HEHA	1,4,7,10,13,16-hexaaazacyclohexadecane-N,N′,N′′,N′′′,N′′′′, N′′′′′-hexaacetic acid
Lpy	(2R)-2-hydroxy-3-[[(S)-hydroxy[[(1S,2R,3R,4S,5S,6R)-2,3,4,5,6-pentahydroxy cyclohexyl]oxy]phosphoryl]oxy]propyl tetradecanoate
